# Shifts in methanogenic community composition and methane fluxes along the degradation of discontinuous permafrost

**DOI:** 10.3389/fmicb.2015.00356

**Published:** 2015-05-12

**Authors:** Susanne Liebner, Lars Ganzert, Andrea Kiss, Sizhong Yang, Dirk Wagner, Mette M. Svenning

**Affiliations:** ^1^Section of Geomicrobiology, GFZ German Research Center for Geosciences, PotsdamGermany; ^2^Department of Experimental Limnology, IGB Leibniz-Institute of Freshwater Ecology and Inland Fisheries, StechlinGermany; ^3^Department of Arctic and Marine Biology, UiT The Arctic University of Norway, TromsøNorway

**Keywords:** discontinuous permafrost, palsa, methane, methanogens, mcrA, peatland succession, climate change

## Abstract

The response of methanogens to thawing permafrost is an important factor for the global greenhouse gas budget. We tracked methanogenic community structure, activity, and abundance along the degradation of sub-Arctic palsa peatland permafrost. We observed the development of pronounced methane production, release, and abundance of functional (*mcrA*) methanogenic gene numbers following the transitions from permafrost (palsa) to thaw pond structures. This was associated with the establishment of a methanogenic community consisting both of hydrogenotrophic (*Methanobacterium*, *Methanocellales*), and potential acetoclastic (*Methanosarcina*) members and their activity. While peat bog development was not reflected in significant changes of mcrA copy numbers, potential methane production, and rates of methane release decreased. This was primarily linked to a decline of potential acetoclastic in favor of hydrogenotrophic methanogens. Although palsa peatland succession offers similarities with typical transitions from fen to bog ecosystems, the observed dynamics in methane fluxes and methanogenic communities are primarily attributed to changes within the dominant *Bryophyta* and *Cyperaceae* taxa rather than to changes in peat moss and sedge coverage, pH and nutrient regime. Overall, the palsa peatland methanogenic community was characterized by a few dominant operational taxonomic units (OTUs). These OTUs seem to be indicative for methanogenic species that thrive in terrestrial organic rich environments. In summary, our study shows that after an initial stage of high methane emissions following permafrost thaw, methane fluxes, and methanogenic communities establish that are typical for northern peat bogs.

## Introduction

The sub-Arctic belongs to the regions where global climate change occurs stronger and earlier than in most other regions of the world (Serreze et al., 2000; Serreze and Francis, 2006). Associated feedbacks with the carbon cycle of the sub-Arctic are of concern considering the large amount of carbon stored in the northern high latitudes primarily in permafrost soils (Tarnocai et al., 2009). The carbon released to the atmosphere as greenhouse gases (GHG) is thereby an important variable for the global climate (IPCC, 2007). Since 2000, total GHG emissions have increased by on average 2.2% per year to 49 Gt in the year 2010 (Victor et al., 2014). Thawing induced changes of the landscape occurs rapidly in sub-Arctic areas of discontinuous and sporadic permafrost (Christensen et al., 2004) such as palsas.

Palsas, frozen peat mounts, typically occur at the margin between the Arctic and sub-Arctic (Seppälä, 1986) and have been largely retreated in the circum-Arctic region since the end of the 20th century mainly as a consequence of rising precipitation (Luoto and Seppälä, 2003). Palsa retreat is thereby often associated with a total collapse of landscape surfaces in the course of less than a decade (Hofgaard, 2004). Palsa ecosystems are thus ideal field model systems to study the response of permafrost thaw upon microbial communities. Permafrost thaw is further associated with an increased release of the potent GHG methane (Schuur et al., 2008; Nauta et al., 2014). Overall, emitted methane contributes 16% to the global GHG emission (Victor et al., 2014). The formation of methane (methanogenesis) is possible at water saturation and low redox conditions and methanogenesis from H_2_/CO_2_ and acetate are the most common pathways in soils. Methane from H_2_/CO_2_ accumulates with lower rates than methane from acetate (Conrad, 1999; Metje and Frenzel, 2007). Methanogenesis includes several enzymatic steps and the last step involving methyl-coenzyme M is a common and conserved trait in all methanogens (Thauer, 1998). Methanogenic communities in northern peatlands have been studied with regard to dominating pathways, community structure, and temperature response. While methanogenesis in acidic peat bogs with low concentrations of mineral salts is mainly attributed to hydrogenotrophic metabolism (Bridgham et al., 2013), low temperatures, nutrient rich conditions, and permafrost environments also promote acetoclastic methanogens (Ganzert et al., 2007; Kotsyurbenko et al., 2007; Barbier et al., 2012; Frank-Fahle et al., 2014). Additionally, methylotrophic methanogenesis via methanol can play a substantial role in certain wetlands (Ganzert et al., 2007; Jiang et al., 2010) where it is formed in the degradation of pectin or lignin (Schink and Zeikus, 1982; Ander and Eriksson, 1985). Through the production of methane, methanogenic archaea (methanogens) of northern peatlands have great significance for methane emissions to the atmosphere and with this for the global climate. Despite much relevant, our understanding on the response of microbial communities of the methane cycle to permafrost thaw is poor (Graham et al., 2011). Two recent studies conducted in a palsa ecosystem from Abisko, northern Sweden, traced methanogenic communities along the thawing of permafrost. There, a single methanogenic species of potentially hydrogenotrophic metabolism was identified as key driver for the formation of methane in a peat bog characterized by permafrost thaw (McCalley et al., 2014; Mondav et al., 2014). While these studies considered methane dynamics until palsa collapse, we aim at extending the knowledge on the methanogenic response to degrading permafrost tracking the methane cycle of an initial permafrost structure (palsa) along complete thaw until late peat bog succession. Assuming that the methanogenic response to permafrost thaw is reflected in alterations of community activity, abundance, and structure we determine these parameters in the light of detailed environmental analyses.

## Materials and Methods

### Site Description and Field Work

The study site was located in the Bøttemyra wetland in Finnmark (northern Norway). Three different geomorphological stages were selected for sampling and analyzing: a degrading palsa (DP; N 69°41.089, E 29°11.951), a thermokarst pond (TP; N 69°41.089, E 29°11.930) next to the degradation front of the palsa and a stabilized depression of a former collapsed palsa (CP; N 69°41.116, E 29°11.752). The TP site was characterized by a dense cover of both sedges and peat mosses while CP was dominated by peat mosses with sparse sedge cover. DP was mainly vegetated with *Empetrum nigrum* and *Rhododendron tomentosum*. All sampling sites were limited in nitrogen with high C/N ratios as well as ammonium and nitrate concentrations below the detection limit (Liebner and Svenning, 2013). Field work and sampling was carried out in July 2011. Methane flux chamber and methane pore water measurements were additionally performed in July 2010.

### Soil Sampling

Three biological replicates were sampled at each site. Sampling at the DP site was carried out using a stainless steel tube (Ø 6 cm) while the TP and CP sites were sampled by cutting three 20 cm × 10 cm blocks out of the peat. Each 10 cm of a core/block was randomly sub-sampled down to 40 cm below the peat surface. All sub-samples from the same depth were combined, transferred into two 15 ml sterile vials, and immediately shock frozen in liquid nitrogen (N_2_) in the field. Long-term storage was at -80°C until further processing.

### Environmental Parameters

Pore water was collected in vertical profiles using small brass tubes as described elsewhere (Liebner et al., 2012). Two biological replicates were sampled. Conductivity, pH, and dissolved oxygen were measured in the field using a multi parameter probe Multi 350i from WTW (Laboratory and Field Products, Nova Analytics). Air and peat temperatures were measured with a hand-held digital thermometer 2000T (Thermocouple Thermometer, Digitron Instrumentation Ltd, England) equipped with a 50 cm long probe. Organic acids (formate, acetate, butyrate) and ethanol were measured by HPLC as described previously (Metje and Frenzel, 2005). Water extractable DOC was determined as NPOC (non-purgable organic carbon) with the Shimadzu TOC–VCPH total organic carbon analyzer.

### Methane Flux, Pore Water Methane Concentrations, and Potential Methane Production

Methane emissions of the field sites were measured in triplicate using plastic chambers sealed with a rubber stopper. The metal frames were pushed into the ground at least one day before the measurements to avoid collecting methane released from the soil during the installation. Water was used in the frames to seal off the chambers from ambient air. Gas chamber samples were collected with a syringe and directly transferred into evacuated 20 ml glass vials. The sampling was done every 6 min for a total period of 30 min. Pore water methane concentrations were measured in triplicates with the exception for site DP where only pore gas could be collected due to the dry conditions. The gas samples were stored in evacuated 20 ml glass vials until analyzed.

Potential methane production from different depths was measured in triplicates. A sample of 6–10 g of soil was transferred to 50 ml serum bottles and 2–10 ml of autoclaved water was added until water saturation was reached. The bottles were sealed with sterile butyl rubber stoppers, the head space was flushed with N_2_/CO_2_ (80:20, v/v) and the samples were incubated at 14°C in the dark. Potential CH_4_ production was additionally determined using H_2_/CO_2_ (80/20 bar) and acetate (20 mM) as substrates. Methane production in the headspace was measured daily for 5 days. All gas samples from the field and laboratory experiments were measured on a gas chromatograph (Agilent 6890 Series, Agilent Technology), and standard gasses were used for calibration.

### DNA Extraction, *mcrA* Gene Amplification, and Sequencing

Soil nucleic acids were extracted in duplicates as described elsewhere (Liebner and Svenning, 2013). The samples were ground in liquid N_2_ to a fine powder. Approximately 0.3 g of sample was mixed with 0.5 ml of extraction buffer (5% cetyltrimethylammonium bromide [CTAB], 120mMK_3_PO_4_ [pH 8]) and subjected to bead beating for 45 s. After phenol-chloroform extraction, nucleic acids were precipitated by incubation with linear acrylamide and 2 volumes of 30% polyethylene glycol 8000 (PEG-8000) for 120 min at room temperature, collected subsequently by centrifugation for 60 min at 4°C, and resuspended in diethyl pyrocarbonate (DEPC)-treated water. Fragments of the *mcrA* gene were amplified using the mlas-mcrA-rev primer pair (Steinberg and Regan, 2008). PCR was carried out in duplicate 25 μl reactions containing 12.5 μl FailSafe PCR 2x PreMix F (Epicentre, Madison, WI, USA), 0.5 μl of each primer (20 μM), 1 U Taq polymerase (Invitrogen, Carlsbad, CA, USA) and 0.5–1 μl 1:5 diluted DNA template, filled up with PCR-grade H_2_O. Reaction conditions were as follows: initial denaturation at 95°C for 5 min, 32 cycles with denaturation at 94°C for 30 s, annealing at 55°C for 45 s, extension at 72°C for 45 s, and a final extension step at 72°C for 10 min. PCR products were checked on a 1% agarose gel with ethidium bromide and purified using a QIAquick Gel Extraction Kit (Qiagen GmbH, Germany). Clone libraries for the *mcrA* gene were established by ligating PCR products into the pCR 2.1–TOPO TA vector and transformed into competent cells *Escherichia coli* TOP10 using the TOPO TA Cloning Kit (Invitrogen) according to the manufacturer’s protocol. White colonies were picked, re-suspended in LB medium containing ampicillin (50 μg ml^-1^) and grown overnight at 37°C. Clones were screened by PCR with vector-specific M13 primers for the correct insert size and a total of 48 amplicons of each sample were sequenced by Macrogen (Amsterdam, Netherlands). The Nucleotide sequences were analyzed with Sequencher (v4.7, Gene Codes, Ann Arbor, MI, USA).

### Bioinformatics and Statistics

Nucleotide sequences were assigned into operational taxonomic units (OTUs) at a cutoff of 0.16 (Yang et al., 2014) using the furthest neighbor clustering method in MOTHUR (Schloss et al., 2009). Phylogenetic assignment and alignment of representative gene sequences was carried out using ARB (Ludwig et al., 2004) and a pre-configured *mcrA* database (http://www.mpi-marburg.mpg.de/downloads/conrad/) that was updated using the CLC sequence viewer software package. The alignment was done using the integrated aligner with subsequent manual refinement. A neighbor-joining tree (Saitou and Nei, 1987) was constructed in ARB with a subset of ~5200 sequences including nearest neighbor and representative isolate sequences. Rarefaction curves were calculated using MOTHUR. The heatmap was constructed based on the OTU abundance table using pheatmap v0.7.4.

### Sequence Accession Numbers

The sequences generated in this study have been deposited in the Genbank nucleotide sequence database under the accession numbers KJ603539 – KJ603857.

### Quantification of 16S rRNA, *mcrA*, and *pmoA* Gene Copies

Quantitative real-time PCR (qPCR) was performed on a CFX96^TM^ cycler from Bio-Rad Laboratories using a SybrGreen assay. We enumerated universal bacteria, and universal archaea gene numbers based on the 16S rRNA gene as well as methanogen and methanotroph genes (*mcrA* and *pmoA*, respectively). Each qPCR run included calibration standards and blanks and was performed in triplicates. Ahead of the final qPCR run, several sample dilutions were tested for potential inhibition. Mostly, a 1:100 dilution was sufficient to exclude inhibition while pronounced inhibition was observed at 1:10 dilutions. We used a final primer concentration of 0.4 μM and 10 μL SsoFast^TM^ EvaGreen supermix (Bio-Rad Laboratories, CA, USA) in 20 μl reactions. The specificity of each run was verified through melt-curve analysis and gel electrophoresis. Bacterial 16S rRNA genes were targeted with the primers Eub341F and Eub534R according to Degelmann et al. (2010) with annealing at 56°C for 20 s. General archaeal 16S rRNA genes were targeted with the primer combination A364 F (Burggraf et al., 1997) and A934 R (Großkopf et al., 1998) with annealing at 55.7°C for 25 s. The *mcrA* gene was amplified with the primers mlas and mcrA-rev (Steinberg and Regan, 2009) with annealing at 55°C for 20 s and *pmoA* genes with the primer combination A189F and A682R (Holmes et al., 1995) with annealing at 60°C for 20 s and data acquisition at 78°C for melting of primer dimers. The potential amplification of nitrifiers using the primer combination A189F and A682R is unlikely given the pronounced limitation in nitrate and ammonium. Moreover, a previous study on methanotrophic community composition and gene expression from the same study sites did not identify any *amoA* like sequences using this primer combination (Liebner and Svenning, 2013).

## Results and Discussion

### The Palsa Ecosystem

**FIGURE 1:**
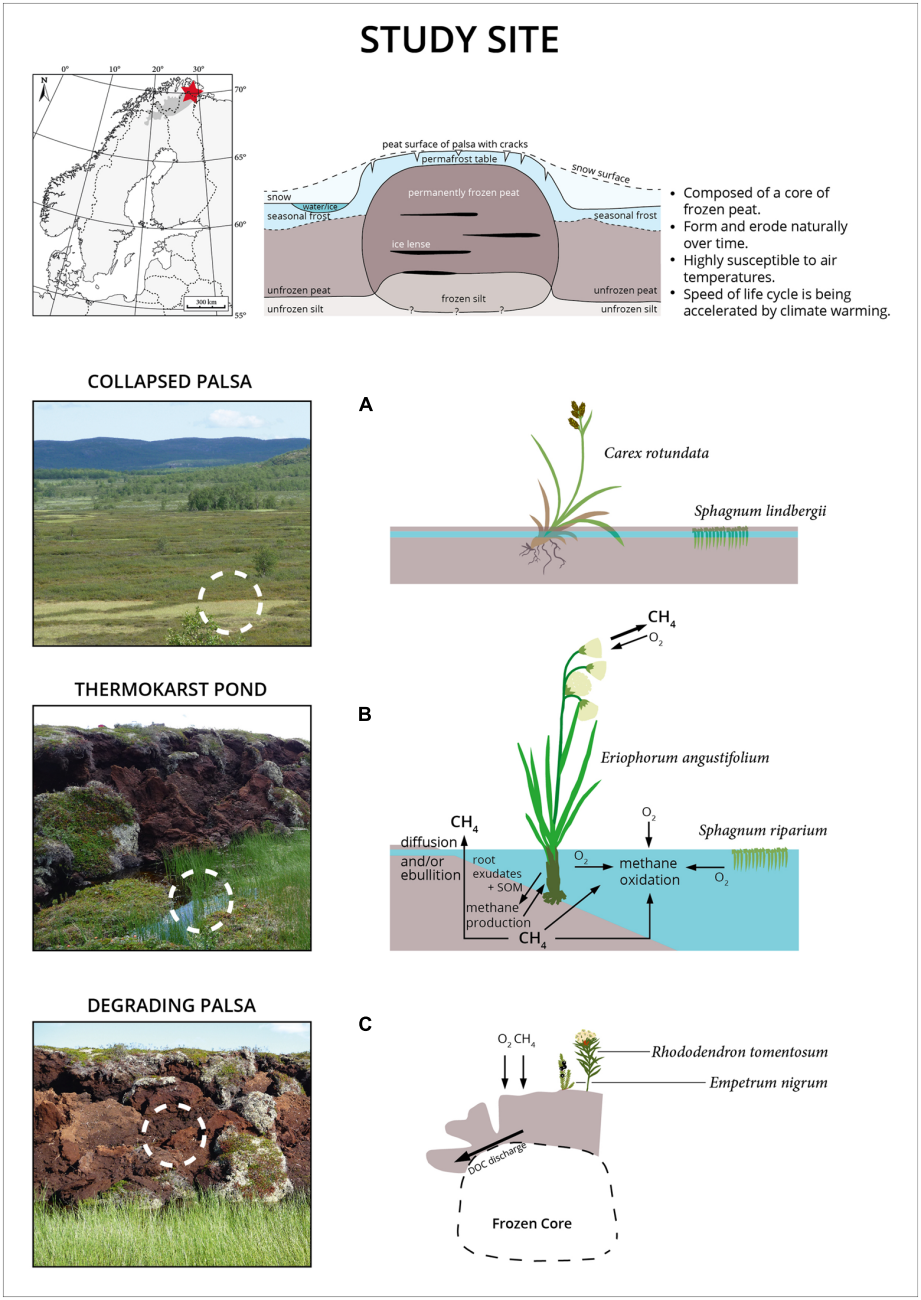
**Location of the study site in northern Norway in Finnmark county (top left), schematic cross section of a palsa (top right) modified from (Seppälä, 1986), and illustration of the sample sites. (A)** Previous collapsed palsa site (CP) which has undergone permafrost degradation. **(B)** Thermokarst pond (TP) structures adjacent to currently degrading palsas (DP), and **(C)** DP. Note the changes in vegetation along palsa degradation and succession. *Sphagnum riparium* typically occurs in pioneer sites with constantly waterlogged conditions while *Sphagnum lindbergii* typically occurs at more stable sites with fluctuating water table.

**FIGURE 2:**
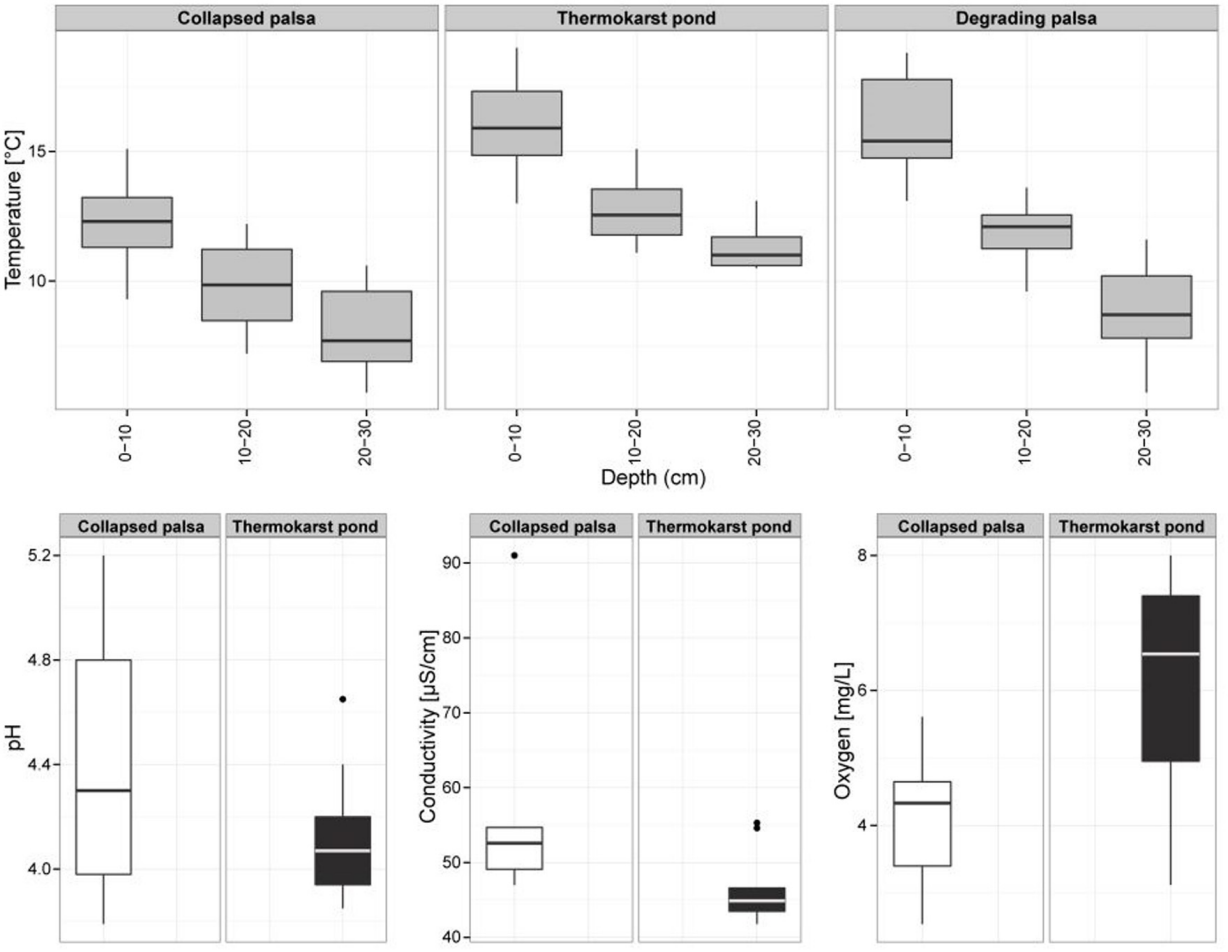
**Environmental parameters (temperature profiles, pH, conductivity, and dissolved oxygen) of the palsa sample sites**.

Palsas are unique climate sensitive permafrost features of high latitude environments. The degradation of palsas leads to a successional gradient including shifts in hydrology, surface morphology, and plant species composition (**Figure 1**). Palsa collapse turned an elevated and dry site (DP) containing an intact ice core into a small freshwater system in the course of less than a decade. The evolving TP is colonized mainly by two plant species, the peat moss *Sphagnum riparium*, floating in large mats, and *Eriophorum angustifolium/russeolum* forming a dense cover of young vascular plants. Complete permafrost thaw and further peat bog succession led to the formation of the CP site, a stable stage within the succession of palsa peatlands. CP is water saturated with a surface layer that can seasonally dry out. *Sphagnum lindbergii* is the dominating plant species sparsely mixed with the sedge *Carex rotundata*. *In situ* temperatures were highest in the freshwater site (TP) where also seasonal temperature variations were low and complete freezing did not occur at any time during the year (data not shown). While pore water pH and conductivity varied little between the two water saturated sites TP and CP, more oxygen was on average dissolved in TP (**Figure 2**). Acetate was 362 (±91) μM in TP and 472 (±252) μM in CP (*n* = 10 for both sites) and formate was 87 (±14) μM and 65 (±50) μM (*n* = 10 for both sites), respectively. The fermentation products ethanol and butyrate were not detected. Acetate and formate concentrations were thus high compared with related studies (Ström et al., 2005; Whitmire and Hamilton, 2008; Liebner et al., 2012) and comparable with other acidic peatlands (Knorr et al., 2008; Küsel et al., 2008). It is well known that acetate accumulates in acidic peatlands although acetoclastic methanogenesis can occur simultaneously in association with plant roots (Bridgham et al., 2013). Also water extractable DOC was comparable between both wet sites although it should be highlighted that DOC values of the top layers of TP stuck out. In detail, DOC ranged between 85 (top) and 26 (bottom) mg L^-1^ in the TP site, between 39 (top) and 27 (bottom) mg L^-1^ in the CP site and between 40 (top) and 27 (bottom) mg L^-1^ in the DP site. DOC values are thus much higher in this palsa ecosystem than in alpine (Liebner et al., 2012; McCalley et al., 2014) and arctic permafrost affected wetlands (King et al., 1998; Wagner et al., 2003) where values did not exceed 25 mg L^-1^. Likely, this results from permafrost thaw and DOC discharge in combination with low pH and water saturation limiting microbial decomposition. In fact, in a simple incubation experiment we observed no significant consumption of DOC in the course of 30 days in either of the sites (Supplementary Table S1) indicating low quality of the DOC.

**FIGURE 3:**
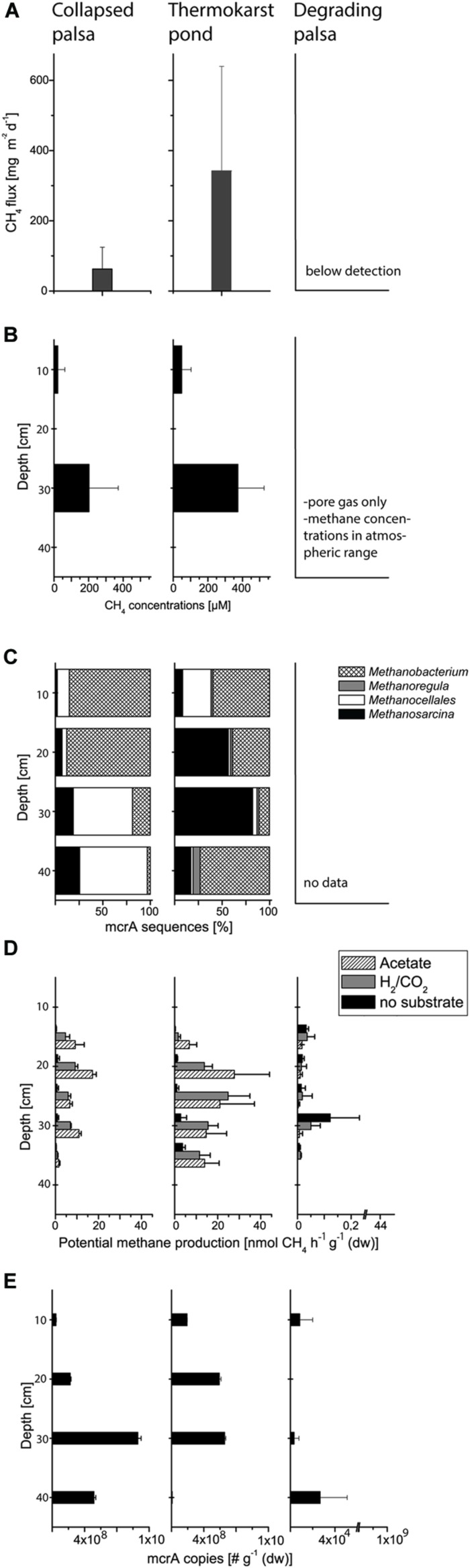
**Methane flux **(A)**, pore water concentrations of methane **(B)**, measured at two depths, 10 and 30 cm, respectively, relative community composition of methanogenic archaea based on *mcrA* gene sequences **(C)**, potential methane production from 15 to 25 cm depth **(D)**, and the number of *mcrA* gene copies **(E)** from the different palsa sampling sites**.

The rapid palsa collapse was associated with the development of a dynamic methane cycle. While no methane emission occurred from the palsa itself, methane emission from TP had mean values of 340 mg (CH_4_) m^-2^ d^-1^ (*n* = 9) with a pronounced spatial and temporal variation reflected in large standard deviations (**Figure 3A**). The fluxes of TP showed thereby notable maxima (up to ~1 g m^-2^ d^-1^) but were on average similar to fluxes from fens in the Alps and the Tibetan Plateau, from the Rocky Mountains, and from wet Arctic tundra (Liebner et al., 2012; and references therein). Toward CP, methane emissions decreased again with mean emissions of ~63 mg (CH_4_) m^-2^ d^-1^ (*n* = 6) which is still higher than methane released from moderately wet Arctic tundra (Sachs et al., 2008; Wille et al., 2008). Methane concentrations in soil pore gas and pore water developed in accordance with the methane fluxes. While concentrations of methane in the palsa itself did not vary from atmospheric values, palsa collapse resulted in pronounced pore water methane concentrations in TP ranging from a mean of 48 μM (*n* = 12) in the uppermost 10 cm to a mean of 374 μM (*n* = 16) between 10 and 40 cm soil depth (**Figure 3B**). Toward CP methane concentrations also decreased reaching a mean value of 22 μM (*n* = 8) in the uppermost 10 cm and a mean of 202 μM (*n* = 11) between 10 and 40 cm soils depth. These values are also similar to related studies from northern and mountain wetlands (Liebner et al., 2012; and references therein).

Summarizing so far, palsa thaw initiates a fast development of intensive methane dynamics while further palsa peatland formation offers similarities with a typical transition from a minerotrophic fen to an ombrotrophic bog and the accompanied changes in methane dynamics (Whiting and Chanton, 1993; Hornibrook and Bowes, 2007). This transition is frequently observed to go along with a shift in methanogenic community structure and physiology from acetoclastic (often in combination with hydrogentrophic) to purely hydrogenotrophic methanogenesis and the accompanied decrease of δ^13^C methane values (Bridgham et al., 2013; McCalley et al., 2014). This shift potentially explains lower methane fluxes as a result of lower methane production efficiency through hydrogenotrophic methanogenesis compared with acetoclastic methanogenesis (Conrad, 1999).

### Methanogen Community Structure

**FIGURE 4:**
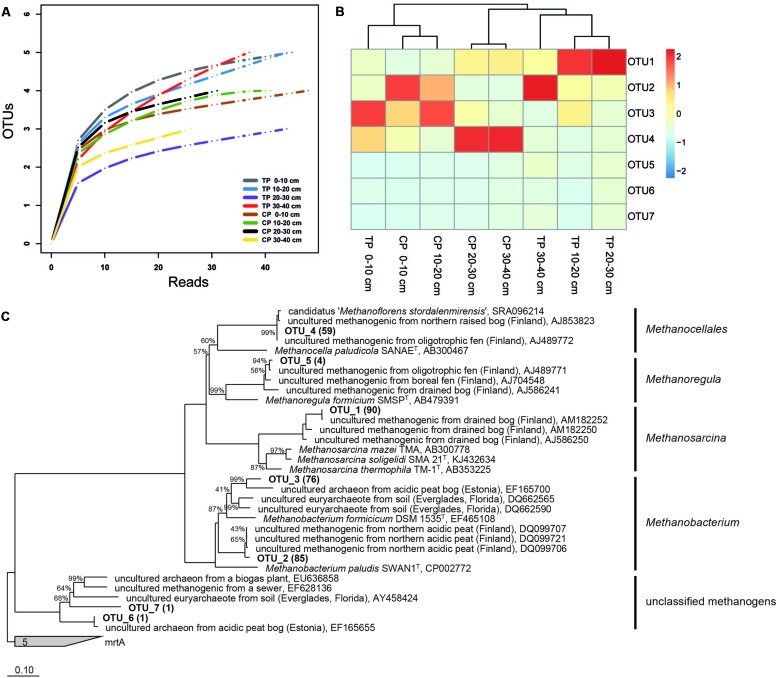
**Rarefaction curves **(A)**, heatmap analysis **(B)**, and phylogenetic tree **(C)** of *mcrA* gene sequences from a sub-Arctic palsa wetland**. In brackets the number of sequences that belong to each OTU. CP, collapsed palsa; TP, thermokarst pond.

Our community data support the shift in methanogen pathways. We identified a methanogenic community clearly structured by both depth and habitat and shifts from a dominance of potential acetoclastic members in TP to a dominance of hydrogenotrophic members in CP (**Figures 3C** and **4A–C**). This shift from acetate to H/CO as the main methanogenic substrate is supported by isotopic values of pore water methane with a δ^13^C-methane of -67.5 ‰ in TP and of -80 ‰ in CP (Frenzel, personal information). Overall, we identified methanogens belonging to *Methanosarcina, Methanobacterium*, *Methanocellales*, and *Methanoregula*. The most abundant OTU_1 belongs to the genus *Methanosarcina* which can use a range of different precursors for methanogenesis. This OTU is representative for the only potentially acetoclastic ‘species’ in our study although we cannot exclude a methylotrophic pathway (Wagner et al., 2013). Thrives in TP in a depth of 10–30 cm characterized by *Eriophorum* roots. *Eriophorum* secretes high amounts of acetate compared to other vascular plants (Saarnio et al., 2004; Ström et al., 2005) and at the same time is suggested to prevent methane oxidation (Frenzel and Rudolph, 1998) either due to absent oxygen supply or massive release of organic acids in the rhizosphere. Acetate was shown to inhibit methane oxidation in peatlands (Wieczorek et al., 2011; Kolb and Horn, 2012) and we also found no indication for methane oxidation in the layers of highest root density in the TP (data not shown). OTU_2 and OTU_3 belong to the hydrogenotrophic genus *Methanobacterium* sp. while OTU_4 is closely related to Candidatus ‘*Methanoflorens stordalenmirensis’* which belongs to the former Rice Cluster I within the order *Methanocellales* and is suggested to use mainly H_2_/CO_2_ as substrate and energy source. This novel methanogen has recently been claimed as key methanogen in thawing permafrost although its biogeography points at a more global and diverse distribution (Conrad et al., 2010; Mondav et al., 2014). In the palsa ecosystem, OTU_4 thrives in the deeper parts of CP (20–40 cm, **Figure 4B**). OTU_5 related to *Methanoregula*, most likely hydrogenotrophic, too, was most abundant in the deeper parts of TP. Rarefaction analysis and OTU calculation indicate a very good coverage of the overall methanogenic diversity (**Figure 4A**) despite the rather low number of *mcrA* sequences. Next generation sequencing would certainly identify numerous rare taxa (less abundant than 1%), however, the dominant methanogenic OTUs were identified. Our community analysis indicates that the palsa methanogenic community is thus not very diverse which is consistent to the recent work of Mondav et al. (2014) in a similar palsa environment. For each of the five most abundant OTUs we also screened the 500 closest hits in GenBank for their isolation source. In all cases there was a preference (OTU_1: 13/14; OTU_2:8/15; OTU_3: 9/17; OTU_4: 15/20; OTU_5: 18/22) toward organic rich, terrestrial habitats which overall points at a very specialized methanogenic community in palsa peatlands. Remaining OTUs had relatively few sequences with OTU_6 and 7 belonging to so far unclassified methanogens clustering with sequences from rumen. The presence of the latter could be due to reindeer grazing.

### Functional Gene Abundance

Based on the enumeration of the *mcrA* gene as functional marker for methanogens we observed no significant differences in methanogenic abundance between TP and CP (**Figure 3E**, Supplementary Figures S1 and S2). Relating *mcrA* gene copy numbers to 16S rRNA copy numbers of all archaea additionally shows that methanogens are dominant among the domain of archaea both in TP and in CP (**Table 1**). Also the abundance of methane oxidizers presented here as *pmoA* gene numbers and in % of 16S rRNA gene copies of all bacteria does not significantly change along the two wet sites where methane oxidizers make up roughly 0.5% of the bacterial community. Nevertheless, the overall contribution of methanogens to methane emissions decreases which is also indicated based on potential rates of methane production (**Figure 3D**). Thus, our study highlights that methanogenic pathway and community structure is more important for soil methane concentrations and fluxes to the atmosphere than total abundance of methanogens and methane oxidizers.

**Table 1:** Copy numbers of bacterial and archaeal 16S rRNA, methanogenic *mcrA*, and methanotrophic *pmoA* genes in three successional stages of a sub-Arctic palsa wetland.

	16S rRNA Bacteria (±SD)	16S rRNA Archaea (±SD)	*mcrA* (±SD)	*pmoA* (±SD)
	**Collapsed palsa (CP)**			
Depth (cm)				
0–10	4.1 × 10^10^ ± 1.8 × 10^9^	3.8 × 10^7^ ± 4.5 × 10^6^	4.5 × 10^7^ ± 3.1 × 10^6^	1.3 × 10^8^ ± 1.0 × 10^7^
10–20	7.8 × 10^10^ ± 2.0 × 10^9^	2.8 × 0^8^ ± 6.1 × 10^6^	2.2 × 10^8^ ± 1.1 × 10^7^	6.9 × 10^8^ ± 3.2 × 10^7^
20–30	5.4 × 10^10^ ± 5.4 × 10^9^	1.7 × 10^9^ ± 5.8 × 10^7^	1.1 × 10^9^ ± 4.1 × 10^7^	1.1 × 10^8^ ± 4.8 × 10^6^
30–40	2.3 × 10^10^ ± 6.5 × 10^8^	8.5 × 10^8^ ± 1.7 × 10^7^	5.2 × 10^8^ ± 2.2 × 10^7^	1.5 × 10^8^ ± 1.4 × 10^7^
				
	**Thermokarst pond (TP)**			
Depth (cm)				
0–10	2.5 × 10^10^ ± 1.1 × 10^9^	1.9 × 10^8^ ± 6.4 × 10^6^	1.9 × 10^8^ ± 3.0 × 10^6^	4.0 × 10^8^ ± 1.3 × 10^7^
10–20	1.9 × 10^10^ ± 3.2 × 10^8^	7.6 × 10^8^ ± 1.6 × 10^7^	5.9 × 10^8^ ± 2.3 × 10^7^	1.1 × 10^8^ ± 7.0 × 10^6^
20–30	2.1 × 10^10^ ± 5.6 × 10^8^	8.3 × 10^8^ ± 2.9 × 10^7^	6.6 × 10^8^ ± 1.3 × 10^7^	2.6 × 10^7^ ± 2.0 × 10^6^
30–40	1.0 × 10^9^ ± 1.4 × 10^7^	4.1 × 10^7^ ± 6.7 × 10^5^	8.5 × 10^6^ ± 1.6 × 10^6^	n.d.
				
	**Degrading palsa (DP)**			
Depth (cm)				
0–10	7.4 × 10^8^ ± 2.6 × 10^7^	5.9 × 10^5^ ± 1.6 × 10^5^	8.3 × 10^3^ ± 1.2 × 10^4^	7.0 × 10^5^ ± 2.0 × 10^4^
10–20	2.1 × 10^9^ ± 9.2 × 10^7^	1.2 × 10^6^ ± 2.1 × 10^5^	n.d.	1.7 × 10^6^ ± 3.7 × 10^4^
20–30	4.0 × 10^8^ ± 2.1 × 10^6^	1.9 × 10^4^ ± 2.4 × 10^4^	3.2 × 10^3^ ± 4.5 × 10^3^	9.1 × 10^5^ ± 4.7 × 10^4^
30–40	2.3 × 10^9^ ± 1.2 × 10^7^	5.6 × 10^4^ ± 6.3 × 10^4^	2.7 × 10^4^ ± 2.4 × 10^4^	2.2 × 10^6^ ± 2.6 × 10^5^

*n.d. – not determined.*

*Values are mean ±SD, *N* = 6 and refer to gram of dry soil.*

## Conclusion

The reason for the change from acetoclastic to hydrogenotrophic methanogenesis in the palsa ecosystem is not easily explained. Related studies attributed this shift to nutrient-poor acidic conditions in ombrogeneous bogs which typically result in a greater abundance of *Sphagnum* sp. and lower net CH_4_ emission rates compared to minerotrophic fens (Ström et al., 2005, 2012). *Sphagnum* mosses in general produce organic compounds that are inhibitory for methanogenesis (Rooney-Varga et al., 2007). Fens possess a higher floral diversity, in particular, vascular vegetation, such as sedges and grasses that release labile carbon to soil via root exudates. Our data show that abundance of *Sphagnum* and sedges as well as nutrient regime and pH alone are insufficient to explain the observed shift in methanogenic community structure and methane fluxes. Both sites are grossly limited in nitrogen and pH values are even lower in the TP site. The amount of organic acids also varied only slightly. We conclude on a major influence of plant species composition on the methane fluxes along permafrost degradation and palsa peatland succession even though our approach is insufficient to provide final evidence on that. Both wet sites, TP and CP, are dominated by *Sphagnum* sp. and sedges, however, of different taxa. The two moss species, *Sphagnum riparium* and *Sphagnum lindbergii*, display differences in cation exchange capacity and the amount of soluble lignin with higher solubility in the case of *Sphagnum lindbergii* (unpublished data). Finally, in contrast to *Carex* sp., *Eriophorum* sp. appear to promote root associated acetoclastic methanogenesis while hampering methane oxidation.

## Conflict of Interest Statement

The authors declare that the research was conducted in the absence of any commercial or financial relationships that could be construed as a potential conflict of interest.
