# Supplementary material for: Shifts in methanogenic community composition and methane fluxes along the degradation of discontinuous permafrost
Source: Front Microbiol. 2015 May 12;6:356. doi: 10.3389/fmicb.2015.00356 (PMC4428212; doi:10.3389/fmicb.2015.00356)
Supplement: Supplementary file 1 [file Data_Sheet_1.PDF]

Table S1.: Incubation experiment of dissolved organic carbon (DOC) retrieved from pore water of the TP and CP sites. DOC was determined as non-purgable organic carbon as described in materials and methods

Sample ID	DOC as NPOC (mg/l)
TP 0d - 1	95.6
TP 0d - 2	94.7
TP 07d - 1	96.8
TP 07d - 2	90.3
TP 14d - 1	93.1
TP 14d - 2	102.2
TP 30d - 1	113.8
TP 30d - 2	92.9
CP 0d - 1	137.1
CP 0d - 2	130.8
CP 7d - 1	133.2
CP 7d - 2	128.8
CP 14d - 1	129.0
CP 14d - 2	131.3
CP 30d - 1	128.3
CP 30d - 2	142.2

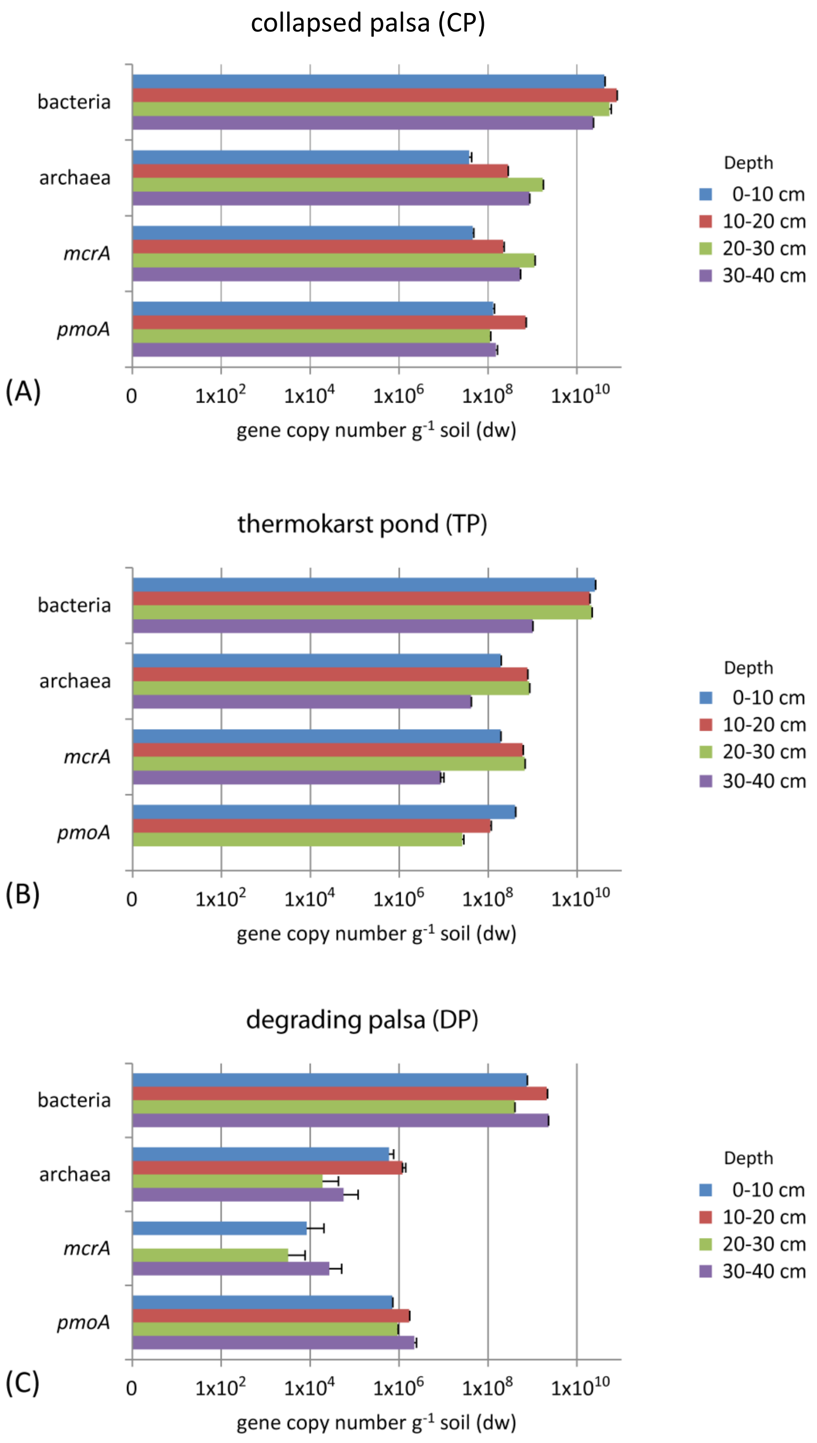

Figure S1: Number of copies of bacterial, archaeal, *mcrA* and *pmoA* genes from: (A) collapsed palsa – CP, (B) thermokarst pond – TP, and (C) degrading palsa - DP. Each gene is grouped separately to show the change with depth, with the x-axis in log scale. Error bars indicate SD.

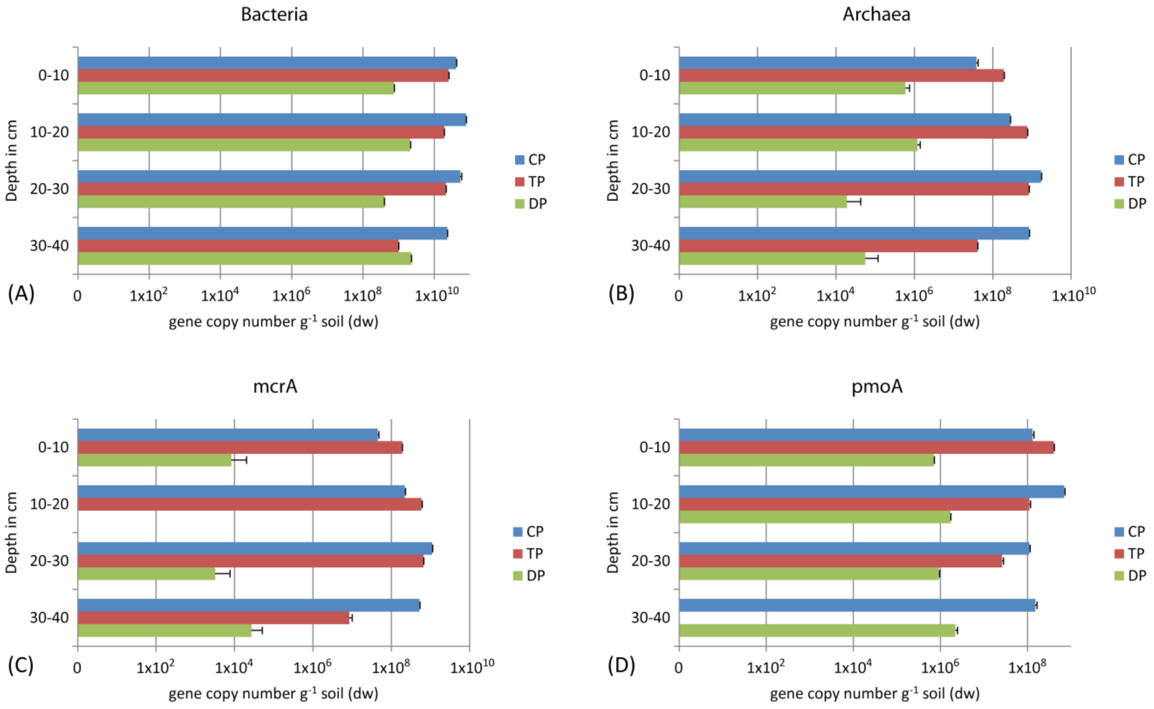

Figure S2: Depth profile of copy numbers for (A) bacteria, (B) archaea, (C) *mcrA* and (D) *pmoA* genes with the sampling sites being combined. CP – collapsed palsa, TP – thermokarst pond, DP – degrading palsa. X-axis in log scale, error bars indicate SD.
